# Large scale clustering of protein sequences with FORCE -A layout based heuristic for weighted cluster editing

**DOI:** 10.1186/1471-2105-8-396

**Published:** 2007-10-17

**Authors:** Tobias Wittkop, Jan Baumbach, Francisco P Lobo, Sven Rahmann

**Affiliations:** 1 Computational Methods for Emerging Technologies, Bielefeld University, Bielefeld, Germany; 2 Genome informatics, Bielefeld University, Bielefeld, Germany; 3 DFG Graduiertenkolleg Bioinformatik, Bielefeld University, Bielefeld, Germany; 4 International Graduate School in Bioinformatics and Genome Research, Center for Biotechnology, Bielefeld, Germany; 5 Laboratorio de Genetica Bioquimica, Universidade Federal de Minas Gerais, Belo Horizonte, Brazil; 6 Bioinformatics for High-Throughput Technologies, Technical University of Dortmund, Germany

## Abstract

**Background:**

Detecting groups of functionally related proteins from their amino acid sequence alone has been a long-standing challenge in computational genome research. Several clustering approaches, following different strategies, have been published to attack this problem. Today, new sequencing technologies provide huge amounts of sequence data that has to be efficiently clustered with constant or increased accuracy, at increased speed.

**Results:**

We advocate that the model of *weighted cluster editing*, also known as *transitive graph projection *is well-suited to protein clustering. We present the FORCE heuristic that is based on transitive graph projection and clusters arbitrary sets of objects, given pairwise similarity measures. In particular, we apply FORCE to the problem of protein clustering and show that it outperforms the most popular existing clustering tools (Spectral clustering, TribeMCL, GeneRAGE, Hierarchical clustering, and Affinity Propagation). Furthermore, we show that FORCE is able to handle huge datasets by calculating clusters for all 192 187 prokaryotic protein sequences (66 organisms) obtained from the COG database. Finally, FORCE is integrated into the corynebacterial reference database CoryneRegNet.

**Conclusion:**

FORCE is an applicable alternative to existing clustering algorithms. Its theoretical foundation, weighted cluster editing, can outperform other clustering paradigms on protein homology clustering. FORCE is open source and implemented in Java. The software, including the source code, the clustering results for COG and CoryneRegNet, and all evaluation datasets are available at .

## Background

### The problem

High-throughput genome sequencing projects have generated massive amounts of DNA and protein sequence data, and will do so more rapidly in the near future. One major challenge continues to be determining protein functions based solely on amino acid sequences. Large-scale pairwise sequence comparison directly results in pairwise similarity measures between protein sequences and is an efficient method to transfer biological knowledge from known proteins to newly sequenced ones. The most widely used method to search for sequence similarities is BLAST [1]. Three challenges arise:

1. Deriving a quantitative similarity measure from the sequence comparison that models homology as well as possible; frequently this is based on the negative logarithm of the BLAST E-value.

2. Inventing a clustering strategy that is sufficiently error-tolerant, since experience shows that sequence similarity alone does not lead to perfect clusterings. A common approach is to use a graph-based model, where proteins are represented as nodes and the similarities as weighted edges.

3. Implementing the chosen clustering strategy efficiently.

We note that many approaches do treat the three challenges separately. In this publication,

1. we use a family of different similarity functions, based on negative logarithms of BLAST E-values and sequence coverage.

2. we show that *weighted graph cluster editing *is an adequate model to identify protein clusters. Weighted graph cluster editing has been recently studied in [2] and is known to be NP-hard.

3. we present a heuristic called FORCE to solve the problem. We show that it provides excellent quality results in practice when compared with an exponential-time exact algorithm, but has a running time that makes it applicable to massive datasets. An extended abstract about the FORCE heuristic, including a comparison to other heuristics, was presented at CSB 2007 [2].

To specify the clustering model, we need the following definition: An undirected simple graph *G *= (*V*, *E*) is called **transitive **if

for all triples *uvw *∈ (V3)
 MathType@MTEF@5@5@+=feaafiart1ev1aaatCvAUfKttLearuWrP9MDH5MBPbIqV92AaeXatLxBI9gBaebbnrfifHhDYfgasaacH8akY=wiFfYdH8Gipec8Eeeu0xXdbba9frFj0=OqFfea0dXdd9vqai=hGuQ8kuc9pgc9s8qqaq=dirpe0xb9q8qiLsFr0=vr0=vr0dc8meaabaqaciaacaGaaeqabaqabeGadaaakeaadaqadaqaauaabeqaceaaaeaacqWGwbGvaeaacqaIZaWmaaaacaGLOaGaayzkaaaaaa@306B@, *uv *∈ *E *and *vw *∈ *E *implies *uw *∈ *E*.

A transitive graph is a union of disjoint cliques, i.e., of complete subgraphs. Each clique represents, in our case, a protein cluster. Since the initial graph, derived from protein similarity values and a similarity threshold, may not be transitive, we need to modify it. This leads to the following computational problems.

#### Graph cluster editing problem (GCEP)

Given an undirected graph *G *= (*V*, *E*), find a transitive graph G* = (*V*, E*), with minimal edge modification distance to *G*, i.e., where |*E *\ E*| + |E* \ *E*| is minimal.

#### Weighted graph cluster editing problem (WGCEP)

To respect the similarity between two proteins, we modify the penalty for deleting and adding edges. First we construct a similarity graph *G *= (*V*, *E*) consisting of a set of objects *V *and a set of edges *E *: = {*uv *∈ (V2)
 MathType@MTEF@5@5@+=feaafiart1ev1aaatCvAUfKttLearuWrP9MDH5MBPbIqV92AaeXatLxBI9gBaebbnrfifHhDYfgasaacH8akY=wiFfYdH8Gipec8Eeeu0xXdbba9frFj0=OqFfea0dXdd9vqai=hGuQ8kuc9pgc9s8qqaq=dirpe0xb9q8qiLsFr0=vr0=vr0dc8meaabaqaciaacaGaaeqabaqabeGadaaakeaadaqadaqaauaabeqaceaaaeaacqWGwbGvaeaacqaIYaGmaaaacaGLOaGaayzkaaaaaa@3069@: *s*(*uv*) > *t*}. Here *s*: (V2)
 MathType@MTEF@5@5@+=feaafiart1ev1aaatCvAUfKttLearuWrP9MDH5MBPbIqV92AaeXatLxBI9gBaebbnrfifHhDYfgasaacH8akY=wiFfYdH8Gipec8Eeeu0xXdbba9frFj0=OqFfea0dXdd9vqai=hGuQ8kuc9pgc9s8qqaq=dirpe0xb9q8qiLsFr0=vr0=vr0dc8meaabaqaciaacaGaaeqabaqabeGadaaakeaadaqadaqaauaabeqaceaaaeaacqWGwbGvaeaacqaIYaGmaaaacaGLOaGaayzkaaaaaa@3069@ → ℝ denotes a similarity function and *t *a user-defined threshold. The resulting cost to add or delete an edge *uv *is set to *cost*(*uv*) : = |*s*(*uv*) - *t*|. The cost to transform a graph *G *= (*V*, *E*) into a graph *G' *= (*V*, *E'*) is consequently defined as *cost*(*G *→ *G'*) : = *cost*(*E *\ *E'*) + *cost*(*E' *\ *E*). As in the GCEP, the goal is to find a transitive graph G* = (*V*, E*), with *cost*(*G *→ G*) = min {*cost*(*G *→ *G'*) : *G' *= (*V*, *E'*) transitive}.

It can be easily seen that the WGCEP is NP-hard, since it is a straightforward generalization of the GCEP, where *s*: (V2)
 MathType@MTEF@5@5@+=feaafiart1ev1aaatCvAUfKttLearuWrP9MDH5MBPbIqV92AaeXatLxBI9gBaebbnrfifHhDYfgasaacH8akY=wiFfYdH8Gipec8Eeeu0xXdbba9frFj0=OqFfea0dXdd9vqai=hGuQ8kuc9pgc9s8qqaq=dirpe0xb9q8qiLsFr0=vr0=vr0dc8meaabaqaciaacaGaaeqabaqabeGadaaakeaadaqadaqaauaabeqaceaaaeaacqWGwbGvaeaacqaIYaGmaaaacaGLOaGaayzkaaaaaa@3069@ → {-1, 1} and *t *= 0. The GCEP has been proved to be NP hard several times, e.g., in [3,4].

### Previous work and novel contributions

There are several approaches to cluster protein families. One of the earliest approaches that took the transitivity concept formally into account was ProClust [5]; however, the concept of editing the graph was not present in this work. The SYSTERS database [6], now at release 4, is based on a set-theoretic SYSTEmatic ReSearching approach and has existed for some time, but seems to have received little updates since early 2005. One of its main features is that it uses family-specific similarity thresholds to define clusters. It does not, however, employ a transitivity concept. In 2006, Paccanaro et al. [7] presented a comparison of the most popular cluster detection methods, like MCL [8], hierarchical clustering [9], GeneRAGE [10], and their own spectral clustering approach, which performs best when evaluated on a subset of the SCOP database. To evaluate our clustering model, we use the same datasets and performance figure. We furthermore include the recently published Affinity Propagation method in our comparison [11]. Additionally, we evaluate our approach against the COG database [12].

The weighted graph cluster editing problem was first considered for protein clustering in our extended abstract [2], where we also introduced the basic idea of the FORCE heuristic.

Here we present a detailed description of the method and an extended parameter estimation procedure using evolutionary training. Our main point in the paper is that the weighted cluster editing problem adequately models the biological homology detection problem if we use appropriate similarity functions and thresholds. The choice of the threshold and similarity function is, of course, critical, and we report the performance for a wide variety of them in the additional files accompanying this paper.

## Methods

### Clustering via graph layouting

We present an algorithm called FORCE that heuristically solves the WGCEP for a connected component and thus for a whole graph. FORCE is motivated by a physically inspired force-based graph layout algorithm developed by Fruchterman and Reingold [13]. The main idea of this approach is to find an arrangement of the vertices in a two-dimensional plane that reflects the edge density distribution of the graph, i.e., vertices from subgraphs with high intra-connecting edge weights should be arranged close to each other and far away from other nodes. This layout is then used to define the clusters by Euclidean single-linkage clustering of the vertices' positions in the plane. To improve the solution, we implemented an additional postprocessing phase. All in all the algorithm proceeds in three main steps: (1) layouting the graph, (2) partitioning, and (3) postprocessing.

#### Layout phase

The goal in this phase is to arrange the vertices in a two-dimensional plane, such that the similarity values are respected. Subsets of nodes with high edge-density should be arranged next to each other, and far away from other nodes. To find a layout that satisfies this criterion, we use a model inspired by physical forces, i.e., nodes can attract and repulse each other. Starting with an initial layout (a circular layout with user defined radius *ρ *and random order), the nodes affect each other depending on their similarity and current position, which leads to a displacement vector for each node and a new arrangement.

Since this model is only inspired by physical forces without friction, it does not include acceleration. For a user-defined number of iterations *R*, the interaction between every pair of nodes and thus the displacement for every node is calculated; then all nodes are simultaneously moved to their new position. We compute the displacements as follows: As described in Algorithm 1 (Appendix), the strength *f*_*u *← *v *_of the effect of one node *v *to another node *u *(i.e., the magnitude of the displacement of *u *caused by *v*) depends on the Euclidean distance *d*(*u*, *v*), on the cost to add or delete the edge and a user defined attraction or repulsion factor *f*_att_, *f*_rep_. More formally,

fu←v={cost(uv)⋅fatt⋅log⁡(d(u,v)+1)|V|for attraction,cost(uv)⋅frep|V|⋅log⁡(d(u,v)+1)for repulsion.
 MathType@MTEF@5@5@+=feaafiart1ev1aaatCvAUfKttLearuWrP9MDH5MBPbIqV92AaeXatLxBI9gBaebbnrfifHhDYfgasaacH8akY=wiFfYdH8Gipec8Eeeu0xXdbba9frFj0=OqFfea0dXdd9vqai=hGuQ8kuc9pgc9s8qqaq=dirpe0xb9q8qiLsFr0=vr0=vr0dc8meaabaqaciaacaGaaeqabaqabeGadaaakeaacqWGMbGzdaWgaaWcbaGaemyDauNaeyiKHWQaemODayhabeaakiabg2da9maaceqabaqbaeaabiGaaaqaamaalaaabaacbiGae83yamMae83Ba8Mae83CamNaemiDaqNaeiikaGIaemyDauNaemODayNaeiykaKIaeyyXICTaemOzay2aaSbaaSqaaiabbggaHjabbsha0jabbsha0bqabaGccqGHflY1cyGGSbaBcqGGVbWBcqGGNbWzcqGGOaakcqWGKbazcqGGOaakcqWG1bqDcqGGSaalcqWG2bGDcqGGPaqkcqGHRaWkcqaIXaqmcqGGPaqkaeaadaabdaqaaiabdAfawbGaay5bSlaawIa7aaaaaeaacqqGMbGzcqqGVbWBcqqGYbGCcqqGGaaicqqGHbqycqqG0baDcqqG0baDcqqGYbGCcqqGHbqycqqGJbWycqqG0baDcqqGPbqAcqqGVbWBcqqGUbGBcqGGSaalaeaadaWcaaqaaiab=ngaJjab=9gaVjab=nhaZjabdsha0jabcIcaOiabdwha1jabdAha2jabcMcaPiabgwSixlabdAgaMnaaBaaaleaacqqGYbGCcqqGLbqzcqqGWbaCaeqaaaGcbaWaaqWaaeaacqWGwbGvaiaawEa7caGLiWoacqGHflY1cyGGSbaBcqGGVbWBcqGGNbWzcqGGOaakcqWGKbazcqGGOaakcqWG1bqDcqGGSaalcqWG2bGDcqGGPaqkcqGHRaWkcqaIXaqmcqGGPaqkaaaabaGaeeOzayMaee4Ba8MaeeOCaiNaeeiiaaIaeeOCaiNaeeyzauMaeeiCaaNaeeyDauNaeeiBaWMaee4CamNaeeyAaKMaee4Ba8MaeeOBa4MaeiOla4caaaGaay5Eaaaaaa@A98C@

Two nodes attract each other if *s*(*uv*) > *t *and repulse each other otherwise. One can see that with increasing distance, attraction strength increases while repulsion strength decreases.

To improve convergence to a stable position with minimal interactions, we added a cooling parameter, also inspired by the algorithm of Fruchterman and Reingold. In our implementation, this means that if the displacement distance exceeds a maximal magnitude *M*_*i *_in iteration *i*, which starts at an initial value *M*_0 _and decreases with every iteration *i*, the movement is limited to it.

The output of this phase is a two-dimensional array *pos *containing the x-y-position of each node. Additional Files 1 and 2 illustrate the layout process and its convergence for two components with 41 and 10 nodes, respectively.

#### Partitioning phase

Using the positions of the vertices from the layout phase, we define clusters by geometric single-linkage clustering, parameterized by a maximal node distance *δ*: As described in Algorithm 2 (Appendix), we start with an arbitrary node *v*_1 _∈ *V *and define a new cluster cv1
 MathType@MTEF@5@5@+=feaafiart1ev1aaatCvAUfKttLearuWrP9MDH5MBPbIqV92AaeXatLxBI9gBaebbnrfifHhDYfgasaacH8akY=wiFfYdH8Gipec8Eeeu0xXdbba9frFj0=OqFfea0dXdd9vqai=hGuQ8kuc9pgc9s8qqaq=dirpe0xb9q8qiLsFr0=vr0=vr0dc8meaabaqaciaacaGaaeqabaqabeGadaaakeaacqWGJbWydaWgaaWcbaGaemODay3aaSbaaWqaaiabigdaXaqabaaaleqaaaaa@30C4@. A node *i *belongs to cv1
 MathType@MTEF@5@5@+=feaafiart1ev1aaatCvAUfKttLearuWrP9MDH5MBPbIqV92AaeXatLxBI9gBaebbnrfifHhDYfgasaacH8akY=wiFfYdH8Gipec8Eeeu0xXdbba9frFj0=OqFfea0dXdd9vqai=hGuQ8kuc9pgc9s8qqaq=dirpe0xb9q8qiLsFr0=vr0=vr0dc8meaabaqaciaacaGaaeqabaqabeGadaaakeaacqWGJbWydaWgaaWcbaGaemODay3aaSbaaWqaaiabigdaXaqabaaaleqaaaaa@30C4@ if there exist nodes *v*_1 _= *i*_0_, ..., *i*_*N *_= *i *∈ *V *with *d*(*i*_*j*_, *i*_*j *+ 1_) ≤ *δ *for all *j *= 0, ..., *N *- 1. Nodes are assigned to cv1
 MathType@MTEF@5@5@+=feaafiart1ev1aaatCvAUfKttLearuWrP9MDH5MBPbIqV92AaeXatLxBI9gBaebbnrfifHhDYfgasaacH8akY=wiFfYdH8Gipec8Eeeu0xXdbba9frFj0=OqFfea0dXdd9vqai=hGuQ8kuc9pgc9s8qqaq=dirpe0xb9q8qiLsFr0=vr0=vr0dc8meaabaqaciaacaGaaeqabaqabeGadaaakeaacqWGJbWydaWgaaWcbaGaemODay3aaSbaaWqaaiabigdaXaqabaaaleqaaaaa@30C4@ until no further nodes satisfy the distance cutoff. Then the next, not yet assigned, node *v*_2 _∈ *V *is chosen to start a new cluster until every node is assigned to some cluster. We denote with Gδ:=∪j=1mcvj
 MathType@MTEF@5@5@+=feaafiart1ev1aaatCvAUfKttLearuWrP9MDH5MBPbIqV92AaeXatLxBI9gBaebbnrfifHhDYfgasaacH8akY=wiFfYdH8Gipec8Eeeu0xXdbba9frFj0=OqFfea0dXdd9vqai=hGuQ8kuc9pgc9s8qqaq=dirpe0xb9q8qiLsFr0=vr0=vr0dc8meaabaqaciaacaGaaeqabaqabeGadaaakeaacqWGhbWrdaWgaaWcbaacciGae8hTdqgabeaakiabcQda6iabg2da9maatadabaGaem4yam2aaSbaaSqaaiabdAha2naaBaaameaacqWGQbGAaeqaaaWcbeaaaeaacqWGQbGAcqGH9aqpcqaIXaqmaeaacqWGTbqBa0GaeSOkIufaaaa@3C6A@ the resulting graph obtained by adding all edges between two nodes of the same cluster and deleting all edges between two nodes of different clusters. To find a good clustering we calculate *cost*(*G *→ *G*_*δ*_) for different *δ*. Starting with *δ *← *δ*_init _: = 0 we increase *δ *by a step size *σ *up to a limit *δ*_max _: = 300.

Experimentation shows that it is beneficial to also increase the step size, i.e. to start with *σ *← *σ*_init _: = 0.01 and increase it by multiplying with a user-defined factor *f*_*σ *_: = 1.1. The solution with lowest cost is returned as the resulting clustering. Algorithm 2 returns the clustering in terms of an *n × n *adjacency matrix E* ∈ {0,1}^*n *× *n*^, and the transformation cost c*.

#### Postprocessing phase

Although the best clustering is not guaranteed to be the optimal one, we often obtain a close to optimal solution in practice. To further improve the results we use a two-step postprocessing heuristic. We denote with *cost*(*C*) the cost to obtain the clustering *C*.

1. To reduce the number of clusters and especially the number of singletons, the first step is to join two clusters if this reduces the overall cost:

Let *C *: = (*c*_1_, ..., *c*_*n*_) be the clustering obtained from the partitioning phase, ordered by size. For all cluster pairs 1 ≤ *i *<*j *≤ *n *we calculate *cost*(*c*_1_, ..., *c*_*i *_∪ *c*_*j*_, ..., *c*_*n*_) until we find a clustering *C' *: = (*c*_1_, ..., *c*_*i' *_∪ *c*_*j'*_, ..., *c*_*n*_) with *cost*(*C'*) <*cost*(*C*). Let (c′1,...,c′n−1)
 MathType@MTEF@5@5@+=feaafiart1ev1aaatCvAUfKttLearuWrP9MDH5MBPbIqV92AaeXatLxBI9gBaebbnrfifHhDYfgasaacH8akY=wiFfYdH8Gipec8Eeeu0xXdbba9frFj0=OqFfea0dXdd9vqai=hGuQ8kuc9pgc9s8qqaq=dirpe0xb9q8qiLsFr0=vr0=vr0dc8meaabaqaciaacaGaaeqabaqabeGadaaakeaadaqadaqaaiqbdogaJzaafaWaaSbaaSqaaiabigdaXaqabaGccqGGSaalcqGGUaGlcqGGUaGlcqGGUaGlcqGGSaalcuWGJbWygaqbamaaBaaaleaacqWGUbGBcqGHsislcqaIXaqmaeqaaaGccaGLOaGaayzkaaaaaa@39F5@ be the sorted vector *C' *. Repeat to attempt joining more clusters until no more join is beneficial.

2. Similar to the Restricted Neighborhood Search Clustering (RNSC [14]), we move a vertex from one cluster to another if this move reduces the overall cost:

As above, let *C *: = (*c*_1_, ..., *c*_*n*_) be the clustering obtained from step 1, ordered by size. For *i*, *j *∈ {1, ..., *n*}, *i *≠ *j*, and every *k *∈ *c*_*i*_, we tentatively move *k *from *c*_*i *_to *c*_*j *_and calculate *cost *(*c*_1_, ..., *c*_*i *_\{*k*}, ..., *c*_*j *_∪ {*k*}, ..., *c*_*n*_), until we find the first such modified clustering with lower cost than *cost*(*C*). We sort the resulting clusters again by size and use them as a new start configuration for the next iteration until no more re-assignments are beneficial.

### Analysis

The worst-case running time of FORCE is given by the addition of those of the three main phases. Layouting runs in Θ (*R*·*n*^2^), where *R *denotes the number of iterations and *n *is the number of nodes in the graph. Since *R *is determined by evolutionary training (see below), it might grow with *n*, but we set an upper bound for *R *to *R*_*max *_= 500 that in practice suffices even for very large datasets.

Partitioning runs in *O*(*D*·*n*^2^), where *D *is the number of different *δ*-values used. This is seen as follows: Each *d*-value requires the construction of an auxiliary graph in *O*(*n*^2^) time, the discovery of its connected components in *O*(|*V*| + |*E*_*δ*_|) = *O*(*n*^2^) time, setting *E' *to the transitive closure of *E*_*δ *_and computing its cost, which is also possible in *O*(*n*^2^) after detecting connected components.

During postprocessing, each iteration takes *O*(*n*^2^) time, since the number of clusters is bonded by *n*. The total time is thus *O*(*P*·*n*^2^), where *P *is the number of postprocessing iterations. While theoretically *P *can grow with *n*, in practice we observe only a small number of iterations until no more improvement occurs.

Thus for all practical purposes, the overall runtime of FORCE is quadratic in the number of nodes.

### Evolutionary parameter training

There are several user-defined parameters to assign, such as the number of iterations *R*, the attraction and repulsion scaling factors *f*_att_, *f*_rep_, the magnitude *M*_0_, and the initial circular layout radius *ρ*. A practical method to find good values is evolutionary training. FORCE implements such a strategy in two different ways.

First, a good parameter combination is determined that can be applied to most of the graphs. This is done during a pre-computation on a training data set. Since, however, the optimal parametser constellation depends on the specific graph, we additionally apply such a training algorithm to each graph. FORCE allows to specify the number of generations to train, and thus to adjust runtime and the quality of the result.

Training works as follows: First we start with a set of 25 randomly generated parameter sets and the initial parameters mentioned above. The parameter sets are sorted by the cost to solve the WGCEP on the given graph. For each generation, we use the best 10 parameter constellations as parents, to generate 15 new combinations. In order to obtain fast convergence to a good constellation, as well as a wide spectrum of different solutions without running into local minima, FORCE splits these 15 new combinations into 3 groups, with 5 members each. The first group consists of parameters obtained only by random combinations of the 10 best already known parameter constellations. The next group is generated with random parameters, while the third group is obtained by a combination of the previous methods. To reduce the runtime for small or very easy to compute solutions, we added a second terminating condition: If at most two different cost appear while calculating the 25 start parameters, the best one is chosen. No more generations are computed.

### Datasets, similarity functions, and parameters

Here we describe the datasets used for the subsequent evaluation. First the ASTRAL dataset from SCOP, as used in [7], is introduced. We also describe a considerably larger dataset obtained from the COG database. BLAST is used for all-against-all similarity searches in all datasets. The similarity measure is a function of the BLAST E-values; we describe three reasonable functions to convert E-values into similarities. The results are used as input for FORCE. All datasets can be downloaded from the FORCE website.

#### SCOP and Astral95

SCOP is an expert, manually curated database that groups proteins based on their 3D structures. It has a hierarchical structure with four main levels (class, fold, superfamily, family). Proteins in the same class have the same type(s) of secondary structures. Proteins share a common fold if they have the same secondary structures in the same arrangement. Proteins in the same superfamily are believed to be evolutionarily related, whereas proteins in the same family exhibit a clear evolutionary relationship [15]. We take the SCOP superfamily classification as ground truth against which we evaluate the quality of a clustering generated by a given algorithm, using reasonable quality measures, such as the F-measure (see below). Since the complete SCOP dataset contains many redundant domains that share a very high degree of similarity, most researchers choose to work with the ASTRAL compendium for sequence and structure analysis in order to generate non-redundant data [16]. ASTRAL allows to select SCOP entries that share no more sequence similarity than a given cutoff, removing redundant sequences.

We extracted two subsets of the ASTRAL dataset of SCOP v1.61 with a cutoff of 95 percent, which means that no two protein sequences share more than 95% of sequence identity. We consider ASTRAL95 as the best possible available reference for remote homology detection on a *structural *basis.

The two subsets are exactly those used in Paccanaro et al.'s work [7]. The first comprises 507 proteins from 6 different SCOP superfamilies, namely *Globin-like*, *EF-hand*, *Cupredoxins*, *(Trans)glycosidases*, *Thioredoxin-like*, and *Membrane all-alpha*. We refer to this dataset as ASTRAL95_1_161.

Due to the fact that SCOP is continuously updated, we decided to evaluate both the original data from [7] (SCOP v1.61) and more recent data from the current SCOP version (SCOP v1.71). The novel version is slightly different. For example, the superfamily *Membrane all-alpha *has been removed in the meantime, and most of its proteins are assigned to different superfamilies. Also, several other proteins have been reassigned to one of the five other superfamilies. This provides another dataset of 589 sequences from the remaining 5 superfamilies, which we refer to as ASTRAL95_1_171.

The second subset consists of 511 sequences from 7 superfamilies, namely *Globin-like*, *Cupredoxins*, *Viral coat and capsid proteins*, *Trypsin-like serine proteases*, *FAD/NAD(P)-binding domain*, *MHC antigen-recognition domain*, and *Scorpion toxin-like*. We refer to this as ASTRAL95_2_161 and ASTRAL95_2_171 respectively. SCOP can be found at [17], while the protein sequences are available at [18].

#### Protein sequences from the COG database

The Cluster of Orthologous Groups (COG) of proteins database is a repository whose main goal is a phylogenetic classification of proteins encoded by complete genomes. It currently consists of 192,187 prokaryotic protein sequences from 66 complete genomes distributed across the three domains of life [12]. COG contains clusters in which at least three individual proteins (or groups of paralogs), originating from three different species, are each other's best BLAST hit in both directions. This strategy is believed to generate clusters of groups of orthologous genes.

We consider COG as the best possible representation of orthology detection, based on *sequence *data alone. We refer to this dataset as the COG dataset. COG can be found [19], while the protein sequences are available at [20].

#### Similarity functions and thresholds

Any attempt to (optimally) solve the WGCEP would be in vain if the target function did not model our goal appropriately. As mentioned earlier, the main challenge is to identify appropriate similarity functions and thresholds. We have used a variety of similarity functions that we describe below.

Assume we are given a set of proteins *V *and a BLAST output file containing multiple high-scoring pairs (HSPs) in both directions. For two proteins *u *and *v *we denote with (*u *← *v*)_*i *_and (*u *→ *v*)_*j*_, where *i *= 1, ..., *k *and *j *= 1, ..., *l*, the corresponding *k *HSPs in one and *l *HSPs in the other direction, respectively.

We consider the following three similarity functions.

#### Best hit (BeH)

This widely used method concentrates on the E-value of a single HSP: For both directions, one looks for the best hit, i.e., the HSP with lowest E-value. To obtain a symmetric similarity function *s*: (V2)
 MathType@MTEF@5@5@+=feaafiart1ev1aaatCvAUfKttLearuWrP9MDH5MBPbIqV92AaeXatLxBI9gBaebbnrfifHhDYfgasaacH8akY=wiFfYdH8Gipec8Eeeu0xXdbba9frFj0=OqFfea0dXdd9vqai=hGuQ8kuc9pgc9s8qqaq=dirpe0xb9q8qiLsFr0=vr0=vr0dc8meaabaqaciaacaGaaeqabaqabeGadaaakeaadaqadaqaauaabeqaceaaaeaacqWGwbGvaeaacqaIYaGmaaaacaGLOaGaayzkaaaaaa@3069@ → ℝ, the negative logarithm of the worst (largest) of the two E-values is taken as similarity measure between *u *and *v*. The resulting symmetric similarity function is then defined as

s(uv):=−log⁡10(max⁡{min⁡i=1,...,kE-value((u←v)i),min⁡j=1,...,lE-value((u→v)j)}).
 MathType@MTEF@5@5@+=feaafiart1ev1aaatCvAUfKttLearuWrP9MDH5MBPbIqV92AaeXatLxBI9gBaebbnrfifHhDYfgasaacH8akY=wiFfYdH8Gipec8Eeeu0xXdbba9frFj0=OqFfea0dXdd9vqai=hGuQ8kuc9pgc9s8qqaq=dirpe0xb9q8qiLsFr0=vr0=vr0dc8meaabaqaciaacaGaaeqabaqabeGadaaakeaacqWGZbWCcqGGOaakcqWG1bqDcqWG2bGDcqGGPaqkcqGG6aGocqGH9aqpcqGHsislcyGGSbaBcqGGVbWBcqGGNbWzdaWgaaWcbaGaeGymaeJaeGimaadabeaakmaabmaabaGagiyBa0MaeiyyaeMaeiiEaG3aaiWabeaadaWfqaqaaiGbc2gaTjabcMgaPjabc6gaUbWcbaGaemyAaKMaeyypa0JaeGymaeJaeiilaWIaeiOla4IaeiOla4IaeiOla4IaeiilaWIaem4AaSgabeaakiabbweafjabb2caTiabbAha2jabbggaHjabbYgaSjabbwha1jabbwgaLnaabmaabaWaaeWaaeaacqWG1bqDcqGHqgcRcqWG2bGDaiaawIcacaGLPaaadaWgaaWcbaGaemyAaKgabeaaaOGaayjkaiaawMcaaiabcYcaSmaaxababaGagiyBa0MaeiyAaKMaeiOBa4galeaacqWGQbGAcqGH9aqpcqaIXaqmcqGGSaalcqGGUaGlcqGGUaGlcqGGUaGlcqGGSaalcqWGSbaBaeqaaOGaeeyrauKaeeyla0IaeeODayNaeeyyaeMaeeiBaWMaeeyDauNaeeyzau2aaeWaaeaadaqadaqaaiabdwha1jabgkziUkabdAha2bGaayjkaiaawMcaamaaBaaaleaacqWGQbGAaeqaaaGccaGLOaGaayzkaaaacaGL7bGaayzFaaaacaGLOaGaayzkaaGaeiOla4caaa@850D@

#### Sum of hits (SoH)

This approach is similar to BeH, but additionally includes every HSP with an E-value smaller than a threshold *m *= 10^-2^. We use this threshold as penalty for every additional HSP. This leads to the similarity function

s(uv):=−log⁡10(max⁡{m−(k−1)⋅∏i=1kE-value((u←v)i),m−(l−1)⋅∏j=1lE-value((u→v)j)}).
 MathType@MTEF@5@5@+=feaafiart1ev1aaatCvAUfKttLearuWrP9MDH5MBPbIqV92AaeXatLxBI9gBaebbnrfifHhDYfgasaacH8akY=wiFfYdH8Gipec8Eeeu0xXdbba9frFj0=OqFfea0dXdd9vqai=hGuQ8kuc9pgc9s8qqaq=dirpe0xb9q8qiLsFr0=vr0=vr0dc8meaabaqaciaacaGaaeqabaqabeGadaaakeaacqWGZbWCcqGGOaakcqWG1bqDcqWG2bGDcqGGPaqkcqGG6aGocqGH9aqpcqGHsislcyGGSbaBcqGGVbWBcqGGNbWzdaWgaaWcbaGaeGymaeJaeGimaadabeaakmaabmaabaGagiyBa0MaeiyyaeMaeiiEaG3aaiWabeaacqWGTbqBdaahaaWcbeqaaiabgkHiTiabcIcaOiabdUgaRjabgkHiTiabigdaXiabcMcaPaaakiabgwSixpaarahabaGaeeyrauKaeeyla0IaeeODayNaeeyyaeMaeeiBaWMaeeyDauNaeeyzau2aaeWaaeaadaqadaqaaiabdwha1jabgcziSkabdAha2bGaayjkaiaawMcaamaaBaaaleaacqWGPbqAaeqaaaGccaGLOaGaayzkaaGaeiilaWIaemyBa02aaWbaaSqabeaacqGHsislcqGGOaakcqWGSbaBcqGHsislcqaIXaqmcqGGPaqkaaGccqGHflY1daqeWbqaaiabbweafjabb2caTiabbAha2jabbggaHjabbYgaSjabbwha1jabbwgaLbWcbaGaemOAaOMaeyypa0JaeGymaedabaGaemiBaWganiabg+GivdGcdaqadaqaamaabmaabaGaemyDauNaeyOKH4QaemODayhacaGLOaGaayzkaaWaaSbaaSqaaiabdQgaQbqabaaakiaawIcacaGLPaaaaSqaaiabdMgaPjabg2da9iabigdaXaqaaiabdUgaRbqdcqGHpis1aaGccaGL7bGaayzFaaaacaGLOaGaayzkaaGaeiOla4caaa@8B61@

#### Coverage (Cov)

The third approach integrates the lengths of a HSP into the similarity function. To determine the coverage, we need the following indicator function:

Iuv(i):={1if in u the position i is covered by any HSP (u←v)n=1,...,k or (u→v)m=1,...,l,0otherwise.
 MathType@MTEF@5@5@+=feaafiart1ev1aaatCvAUfKttLearuWrP9MDH5MBPbIqV92AaeXatLxBI9gBaebbnrfifHhDYfgasaacH8akY=wiFfYdH8Gipec8Eeeu0xXdbba9frFj0=OqFfea0dXdd9vqai=hGuQ8kuc9pgc9s8qqaq=dirpe0xb9q8qiLsFr0=vr0=vr0dc8meaabaqaciaacaGaaeqabaqabeGadaaakeaatuuDJXwAK1uy0HMmaeHbfv3ySLgzG0uy0HgiuD3BaGabaiab=Hi8jnaaBaaaleaacqWG1bqDcqWG2bGDaeqaaOGaeiikaGIaemyAaKMaeiykaKIaeiOoaOJaeyypa0ZaaiqabeaafaqaaeGacaaabaGaeGymaedabaGaeeyAaKMaeeOzayMaeeiiaaIaeeyAaKMaeeOBa4MaeeiiaaIaemyDauNaeeiiaaIaeeiDaqNaeeiAaGMaeeyzauMaeeiiaaIaeeiCaaNaee4Ba8Maee4CamNaeeyAaKMaeeiDaqNaeeyAaKMaee4Ba8MaeeOBa4MaeeiiaaIaemyAaKMaeeiiaaIaeeyAaKMaee4CamNaeeiiaaIaee4yamMaee4Ba8MaeeODayNaeeyzauMaeeOCaiNaeeyzauMaeeizaqMaeeiiaaIaeeOyaiMaeeyEaKNaeeiiaaIaeeyyaeMaeeOBa4MaeeyEaKNaeeiiaaIaeeisaGKaee4uamLaeeiuaaLaeeiiaaIaeiikaGIaemyDauNaeyiKHWQaemODayNaeiykaKYaaSbaaSqaaiabd6gaUjabg2da9iabigdaXiabcYcaSiabc6caUiabc6caUiabc6caUiabcYcaSiabdUgaRbqabaGccqqGGaaicqqGVbWBcqqGYbGCcqqGGaaicqGGOaakcqWG1bqDcqGHsgIRcqWG2bGDcqGGPaqkdaWgaaWcbaGaemyBa0Maeyypa0JaeGymaeJaeiilaWIaeiOla4IaeiOla4IaeiOla4IaeiilaWIaemiBaWgabeaakiabcYcaSaqaaiabicdaWaqaaiabb+gaVjabbsha0jabbIgaOjabbwgaLjabbkhaYjabbEha3jabbMgaPjabbohaZjabbwgaLjabc6caUaaaaiaawUhaaaaa@ACB7@

The coverage can now be defined as

coverage(uv):=min⁡(1|u|∑i=1|u|Iuv(i),1|v|∑i=1|v|Ivu(i)).
 MathType@MTEF@5@5@+=feaafiart1ev1aaatCvAUfKttLearuWrP9MDH5MBPbIqV92AaeXatLxBI9gBaebbnrfifHhDYfgasaacH8akY=wiFfYdH8Gipec8Eeeu0xXdbba9frFj0=OqFfea0dXdd9vqai=hGuQ8kuc9pgc9s8qqaq=dirpe0xb9q8qiLsFr0=vr0=vr0dc8meaabaqaciaacaGaaeqabaqabeGadaaakeaacqqGJbWycqqGVbWBcqqG2bGDcqqGLbqzcqqGYbGCcqqGHbqycqqGNbWzcqqGLbqzcqGGOaakcqWG1bqDcqWG2bGDcqGGPaqkcqGG6aGocqGH9aqpcyGGTbqBcqGGPbqAcqGGUbGBdaqadaqaamaalaaabaGaeGymaedabaWaaqWaaeaacqWG1bqDaiaawEa7caGLiWoaaaWaaabCaeaatuuDJXwAK1uy0HMmaeHbfv3ySLgzG0uy0HgiuD3BaGabaiab=Hi8jnaaBaaaleaacqWG1bqDcqWG2bGDaeqaaOGaeiikaGIaemyAaKMaeiykaKIaeiilaWcaleaacqWGPbqAcqGH9aqpcqaIXaqmaeaadaabdaqaaiabdwha1bGaay5bSlaawIa7aaqdcqGHris5aOWaaSaaaeaacqaIXaqmaeaadaabdaqaaiabdAha2bGaay5bSlaawIa7aaaadaaeWbqaaiab=Hi8jnaaBaaaleaacqWG2bGDcqWG1bqDaeqaaOGaeiikaGIaemyAaKMaeiykaKcaleaacqWGPbqAcqGH9aqpcqaIXaqmaeaadaabdaqaaiabdAha2bGaay5bSlaawIa7aaqdcqGHris5aaGccaGLOaGaayzkaaGaeiOla4caaa@7F9D@

In order to obtain a good similarity function, we control the influence of the coverage on the overall similarity function by a user-defined factor *f*, and set

*s*(*uv*) : = *s'*(*uv*) + *f *·coverage(*uv*).

Here *s' *: (V2)
 MathType@MTEF@5@5@+=feaafiart1ev1aaatCvAUfKttLearuWrP9MDH5MBPbIqV92AaeXatLxBI9gBaebbnrfifHhDYfgasaacH8akY=wiFfYdH8Gipec8Eeeu0xXdbba9frFj0=OqFfea0dXdd9vqai=hGuQ8kuc9pgc9s8qqaq=dirpe0xb9q8qiLsFr0=vr0=vr0dc8meaabaqaciaacaGaaeqabaqabeGadaaakeaadaqadaqaauaabeqaceaaaeaacqWGwbGvaeaacqaIYaGmaaaacaGLOaGaayzkaaaaaa@3069@ → ℝ denotes one of the previously presented similarity functions, BeH or SoH.

#### Parameter choices

The initial parameters obtained from the pre-processing training are *R *= 186, *f*_att _= 1.245, *f*_rep _= 1.687, *M*_0 _= 633, and *ρ *= 200 for the protein clustering problem. Furthermore, we apply evolutionary training to each problem instance, as described in the Algorithms section.

## Results

This section contains three different types of results. First we discuss the appropriateness of the WGCEP model for the detection of clusters of homologous proteins using the ASTRAL dataset described earlier. Next we show that the FORCE heuristic is fast in practice, and compares favorably against an exact (exponential-time) fixed-parameter algorithm in terms of solution quality. We show that FORCE is able to handle very large datasets efficiently, in particular the COG dataset described previously. Finally, we have integrated the clustering results of FORCE into the corynebacterial reference database CoryneRegNet [21,22].

### Evaluation of the WGCEP model

To show that the WGCEP model is adequate for protein homology clustering, we evaluate our algorithm in the same way as Paccanaro et al. did in their article [7], using the so-called F-measure to quantify the agreement of FORCE's result with the reference clustering provided by the ASTRAL dataset.

We first explain the F-measure, which equally combines precision and recall. Let *K *= (*K*_1_, ..., *K*_*m*_) be the clustering obtained from the algorithm and *C *= (*C*_1_, ..., *C*_*l*_) the reference clustering. Furthermore, we denote with *n *the total number of proteins and with *n*_*i*_, *n*^*j *^the number of proteins in the cluster *K*_*i *_and *C*_*j*_, respectively. Following this, nij
 MathType@MTEF@5@5@+=feaafiart1ev1aaatCvAUfKttLearuWrP9MDH5MBPbIqV92AaeXatLxBI9gBaebbnrfifHhDYfgasaacH8akY=wiFfYdH8Gipec8Eeeu0xXdbba9frFj0=OqFfea0dXdd9vqai=hGuQ8kuc9pgc9s8qqaq=dirpe0xb9q8qiLsFr0=vr0=vr0dc8meaabaqaciaacaGaaeqabaqabeGadaaakeaacqWGUbGBdaqhaaWcbaGaemyAaKgabaGaemOAaOgaaaaa@30F6@ is the number of proteins in the intersection *K*_*i *_∩ *C*_*j*_. The F-measure is defined as

F(K,C):=1n∑j=1lnj⋅max⁡1≤i≤m(2nijni+nj).
 MathType@MTEF@5@5@+=feaafiart1ev1aaatCvAUfKttLearuWrP9MDH5MBPbIqV92AaeXatLxBI9gBaebbnrfifHhDYfgasaacH8akY=wiFfYdH8Gipec8Eeeu0xXdbba9frFj0=OqFfea0dXdd9vqai=hGuQ8kuc9pgc9s8qqaq=dirpe0xb9q8qiLsFr0=vr0=vr0dc8meaabaqaciaacaGaaeqabaqabeGadaaakeaacqWGgbGrcqGGOaakcqWGlbWscqGGSaalcqWGdbWqcqGGPaqkcqGG6aGocqGH9aqpdaWcaaqaaiabigdaXaqaaiabd6gaUbaadaaeWbqaaiabd6gaUnaaCaaaleqabaGaemOAaOgaaaqaaiabdQgaQjabg2da9iabigdaXaqaaiabdYgaSbqdcqGHris5aOGaeyyXIC9aaCbeaeaacyGGTbqBcqGGHbqycqGG4baEaSqaaiabigdaXiabgsMiJkabdMgaPjabgsMiJkabd2gaTbqabaGcdaqadaqaamaalaaabaGaeGOmaiJaemOBa42aa0baaSqaaiabdMgaPbqaaiabdQgaQbaaaOqaaiabd6gaUnaaBaaaleaacqWGPbqAaeqaaOGaey4kaSIaemOBa42aaWbaaSqabeaacqWGQbGAaaaaaaGccaGLOaGaayzkaaGaeiOla4caaa@5D2B@

As mentioned earlier, Paccanaro et al. previously compared the most popular protein clustering tools against their own spectral clustering: GeneRAGE, TribeMCL, and Hierarchical clustering. Since there is no need to replicate existing results, we use the same data (ASTRAL95_1_161 and ASTRAL95_2_161). Table 1 summarizes the results: Using FORCE, we obtain slightly better agreements than with spectral clustering. The best similarity function parameters and score threshold for the ASTRAL95_1_161 dataset were Cov-scoring using *f *= 20 and BeH as a secondary scoring function, and *t *= *-*2.2. For the ASTRAL95_2_161 dataset, this was Cov-scoring with *f *= 19 and SoH as secondary scoring function with *t *= *-*1.6.

**Table 1:** Evaluation of protein clustering tools. The F-measure (between 0 and 1) measures the agreement between a clustering resulting from a given algorithm and a reference clustering provided with the dataset. An F-measure of 1 indicates perfect agreement. ASTRAL95_1_161 and ASTRAL95_2_161 refer to the two datasets of SCOP v1.61 used by Paccanaro et al. for spectral clustering [7]. All reported values, except for our algorithm FORCE and for Affinity Propagation, are from the same reference.

Dataset	Method	F-measure
ASTRAL95_1_161	FORCE	0.85
ASTRAL95_1_161	Spectral clustering	0.81
ASTRAL95_1_161	Affinity Propagation	0.65
ASTRAL95_1_161	GeneRAGE	0.47
ASTRAL95_1_161	TribeMCL	0.32
ASTRAL95_1_161	Hierarchical clustering	0.26

ASTRAL95_2_161	FORCE	0.89
ASTRAL95_2_161	Spectral clustering	0.82
ASTRAL95_2_161	Affinity Propagation	0.69
ASTRAL95_2_161	GeneRAGE	0.54
ASTRAL95_2_161	TribeMCL	0.52
ASTRAL95_2_161	Hierarchical clustering	0.42

Note that in the present context, we do not consider it as cheating to optimize the similarity function and threshold: We want to check how far the WGCEP model can retrieve the biologically correct clustering under ideal conditions. The same kind of optimization was applied by Paccanaro et al. in [7]. Table 1 also shows the F-measures for the Affinity Propagation (AP) approach, which was recently published in [11]. We used the same data and also varied necessary input parameters to evaluate against the best possible performance of AP. For ASTRAL95_1_161, this was Cov-scoring with *f *= 20 and SoH as secondary scoring function with fixed preference *pre *= 600, and damping factor *df *= 0.8. For ASTRAL95_2_161, this was Cov-scoring with *f *= 14 and SoH as secondary scoring function with *pre *= 600, and *df *= 0.75. For both datasets, AP performs worse than Spectral clustering.

**Figure 1:**
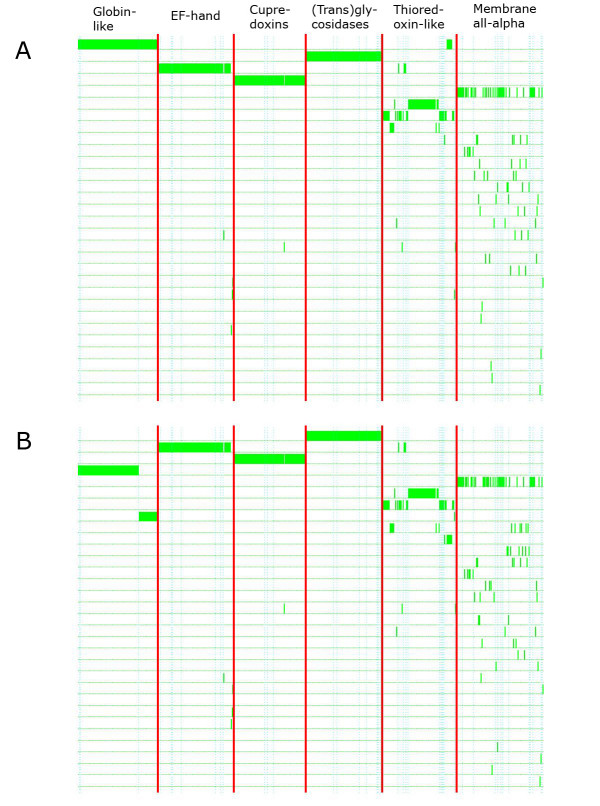
Graphical summary of the obtained clustering results of FORCE for the two similarity functions (A) BeH and (B) SoH, and dataset ASTRAL95_1_161. We used MATLAB scripts provided by Paccanaro to create images similar to those of Figure 3 in [7]. Each row corresponds to a cluster. Green bars represent a protein assignment to a cluster; each protein is present in only one of the clusters. Boundaries between superfamilies are shown by vertical red lines, and boundaries between families within each superfamily are shown by dotted blue lines.

Figure 1 exemplarily illustrates the obtained clustering results for two similarity functions, and dataset ASTRAL95_1_161. One can see that the classification is very good for the superfamilies *Globin-like*, *EF-hand*, *Cupredoxins*, *(Trans)glycosidases*. *Thioredoxin-like *and *Membrane all-alpha *are split into several clusters. Note, that for *Globin-like *(left column) using similarity function SoH (B), the superfamily is split into two clusters, where the second (the lower one) represents a family.

We generated images in the same style for all datasets, zipped them, and provide them as Additional File 3. We additionally evaluate FORCE with the newest ASTRAL95 datasets (ASTRAL95_1_171 and ASTRAL95_2_171). Table 2 shows the resulting F-measures for a variety of similarity functions and parameter choices. All of these achieve higher F-measures than Spectral clustering, or AP.

**Table 2:** Evaluation of the WGCEP model. The best F-measures for each dataset and each similarity function. ASTRAL95_1_161 and ASTRAL95_2_161 are as in Table 1. ASTRAL95_1_171 and ASTRAL95_2_171 refer to the updated ASTRAL95 data of SCOP v1.71. BeH or SoH denote the similarity function, while the coverage factor *f *represents the influence of the coverage to the similarity.

Dataset	Similarity	Factor *f*	Threshold	F-measure
ASTRAL95_1_171	SoH	18	-3.0	0.91
ASTRAL95_1_171	BeH	15	-3.4	0.90
ASTRAL95_2_161	SoH	19	-1.6	0.89
ASTRAL95_2_171	SoH	15	-3.2	0.88
ASTRAL95_2_161	BeH	14	-2.4	0.87
ASTRAL95_2_171	BeH	13	-2.6	0.85
ASTRAL95_1_161	BeH	20	-2.2	0.85
ASTRAL95_1_161	SoH	20	-1.8	0.83

In Additional File 4, we provide F-measures of FORCE for a wide range of thresholds and coverage factors, for all used datasets and similarity functions. Good clustering quality is also reached by using other thresholds and similarity measures for all test datasets. In Additional File 5, we give F-measures for a range of thresholds, but with fixed coverage factor *f *= 20, for dataset ASTRAL95_1_161, and similarity function BeH. In Additional File 6, we provide F-measures for Affinity Propagation for a wide range of parameters and coverage factors, for all used datasets and similarity functions.

### Quality and running time of the heuristic

After evaluating the WGCEP as a reasonable clustering paradigm, we address the performance of the FORCE heuristic: We compare the running time and solution quality against a slow exact algorithm on the large COG dataset. A recently developed fixed-parameter (FP) algorithm for the WGCEP [2] extends ideas of previously developed FP algorithms for the (unweighted) GCEP by Gramm et al. [23,24] and Dehne et al. [25], and has a running time of *O*(3^*k *^+ |*V|*^3 ^log |*V|*), if there exists a transitive projection of cost at most *k*. This allows us to find the optimal solution for a WGCEP, given a graph *G *= (*V*, *E*) up to size *|V| *≈ 50 in appropriate time. To our knowledge, the implementation of this algorithm is the fastest available exact WGCEP solving program.

**Figure 2:**
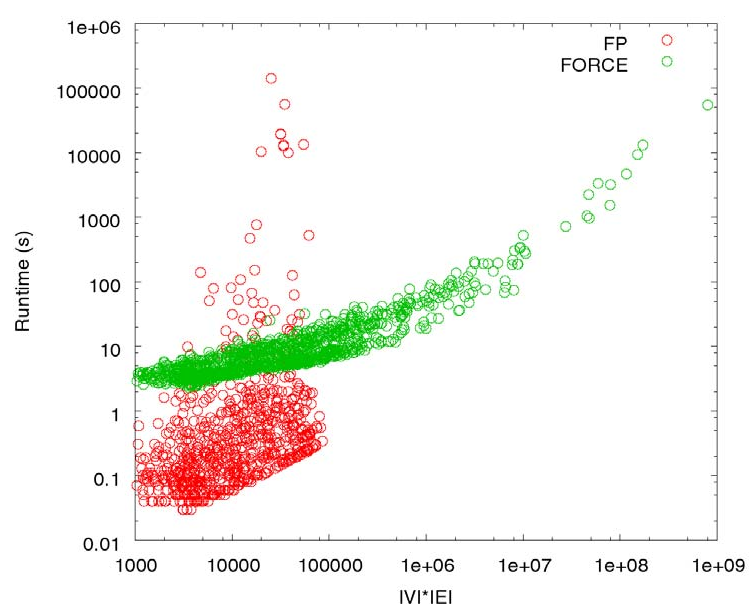
Comparison of the running times of FORCE against the exact fixed-parameter algorithm described in [2]. Plotted is the running time (y-axis in seconds) for different graph sizes (x-axis). Solely for visualization purposes, we describe the size of a graph on the x-axis as |*V*|·|*E*|. All graphs have been constructed from prokaryotic COG protein sequence comparisons using BeH as scoring function. Note that both axes are scaled logarithmically. The red points correspond to FORCE running times, and the blue points to the FP algorithm, respectively.

In order to compare the two approaches we use the COG dataset, split into connected subgraphs using similarity function SoH and a threshold of 10. We extracted 1244 connected components (with *|V| *≤ 3 387). For the evaluation, we restricted the maximal run time to 48 hours. The FP algorithm thus could only be applied to 825 components with *|V| *≤ 56. For the remaining components, the FP algorithm was terminated unsuccessfully after 48 hours. Due to the large number of graphs, we abstained from applying FP to graphs with *|V| *≥ 100, because it is very likely that runtime would exceed 48 hours. Figure 2 illustrates a running time comparison of the FP (blue) and the heuristic algorithm (red). FORCE has been configured to use one generation of evolutionary parameter training for each graph, as described in the Algorithms section. All time measurements were taken on a SunFire 880 with 900 MHz UltraSPARC III+ processors and 32 GB of RAM.

**Figure 3:**
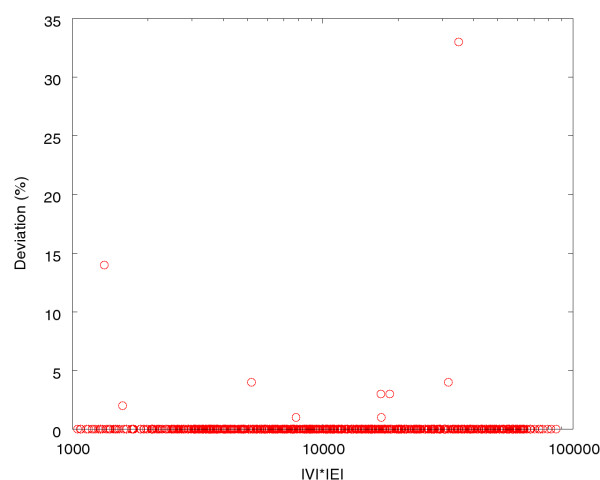
Relative cost deviations (y-axis in %) of the FORCE solutions from the optimal solutions found by the exact fixed-parameter algorithm described in [2]. The x-axis is as in Figure 2 (logarithmically scaled).

One can see that for large graphs *|V|*·*|E| *≥ 100 000), FORCE is much faster than the exact FP algorithm. Note that the axes are logarithmically scaled. We evaluate the quality of the FORCE heuristic by comparing the relative cost increase of the reported solution, with respect to the provably optimal solution. For 814 out of the 825 comparable components, the heuristic determines the optimal solution. The optimal cost over all 825 components is 171 986.8, while FORCE finds a solution with a total cost of 172 244.6, which is a difference of 0.15%. Figure 3 illustrates these numbers. Note that most of the data points lie on the x-axis and hence indicate that the optimal solution was found.

In addition to the direct running time and quality comparison, we make all connected components and clustering results of the COG dataset available on the FORCE website, using the following similarity functions and thresholds: BeH/10, BeH/20, SoH/10, SoH/20. These choices do not reproduce the original COG clustering; we obtain the following F-measures: 0.64 (BeH/10), 0.56 (BeH/20), 0.61 (SoH/10), and 0.53 (SoH/20). It should be noted that a) the COG clustering problem has very different properties than the SCOP clustering problem, and b) here we have not optimized in any way the scoring function and threshold. We discuss this further below.

### CoryneRegNet

CoryneRegNet (online available at [26]) allows a pertinent data management of regulatory interactions along with the genome-scale reconstruction of transcriptional regulatory networks of corynebacteria relevant in human medicine and biotechnology, together with *Escherichia coli*. CoryneRegNet is based on a multi-layered, hierarchical and modular concept of transcriptional regulation and was implemented with an ontology-based data structure. It integrates the fast and statistically sound method PoSSuMsearch [27] to predict transcription factor binding sites within and across species. Reconstructed regulatory networks can be visualized on a web interface and as graphs. Special graph layout algorithms have been developed to facilitate the comparison of gene regulatory networks across species and to assist biologists with the evaluation of predicted and graphically visualized networks in the context of experimental results. To extend the comparative features, we need adequate data on gene and protein clusters. The integration of this information would widen the scope of CoryneRegNet and assist the user with the reconstruction of unknown regulatory interactions [21,22].

Using FORCE, we calculated protein clusters for all organisms integrated in CoryneRegNet: *Corynebacterium diphtheriae*, *Corynebacterium efficiens*, *Corynebacterium glutamicum*, *Corynebacterium jeikeium*, *Escherichia coli*, *Mycobacterium tuberculosis CDC1551 *and *Mycobacterium tuberculosis H37Rv *(altogether 22,797 proteins). Based on cluster size distribution, we empirically determined a comparatively high threshold of 30 (which can be explained by the relatively close evolutionary relationship of most organisms in CoryneRegNet) and similarity function SoH to create the FORCE input files based on the all-vs-all BLAST results that are generated during CoryneRegNet's data warehousing process.

The results computed by FORCE are parsed into the object oriented back-end and further on translated into the ontology based data structure of CoryneRegNet. We added a new concept class FORCECluster and a relation type b_fc (belongs to FORCECluster), which links the proteins to their clusters. Finally, we adapted the CoryneRegNet back-end to import the new data into the database and the web-front-end to present the clusters.

## Discussion and Conclusion

We have shown that the WGCEP is an adequate model for remote protein homology clustering from sequence-based similarity measures and can outperform existing clustering approaches. Part of this effect is certainly attributable to the class of similarity functions that we consider. Nevertheless, in this particular application, the WGCEP paradigm (or rather, our implementation) even outperforms the Affinity Propagation approach, for which we use the same class of similarity functions and a similar parameter optimization as for our approach.

We described FORCE, a heuristic algorithm for the NP-hard weighted graph cluster editing problem. Compared to the currently most efficient exact (exponential-time) fixed-parameter algorithm for this problem, we have demonstrated empirically that FORCE regularly provides solutions that are optimal, although no guarantee is given by the algorithm. In contrast to the exact algorithm, FORCE can solve the problem for graphs with several thousands of nodes in reasonable time.

One of our motivations to develop a rapid and high-quality clustering algorithm arose from the need to extend the data warehouse CoryneRegNet with protein family information. Consequently, the clustering derived by FORCE has been integrated into the system.

We emphasize that FORCE can cluster any set of objects connected by any kind of similarity function using the concept of editing a graph into a transitive graph with minimum cost changes. The integrated evolutionary parameter training method ensures good performance on any kind of data.

Several issues remain to be resolved with the cluster editing or transitive projection approach. One disadvantage of the method is that it uses the same threshold for all clusters to determine the cost of adding or removing edges. The authors of SYSTERS [6] report an interesting approach to choose thresholds in a dynamical way. Finding a way of incorporating dynamic thresholds into cluster editing would certainly enhance its applicability.

The other issue we need to discuss is more global and applies to any clustering algorithm and concerns the choice of parameters. For evaluating the WGCEP model with the SCOP datasets, we have optimized similarity function and threshold (the "parameters") by using the known truth as a reference and thus determined that there exists a (reasonably simple) similarity function that models the truth rather well. In practice, given an unknown dataset, we do not know which parameters lead to the unknown truth. Therefore we need to find properties of the resulting clustering (beyond the target function) that tell us something about the quality of the clustering. For CoryneRegNet, we were able to use the cluster size distribution, as we had expert biological support. In other cases, it is an open challenge to find properties of the clustering that can be easily verified by knowledgeable experts in the field.

## Availability and Requirements

Project name: FORCE

Project home page: 

Operating system(s): Platform independent

Programming language: Java 6

License: Academic Free License (AFL)

Any restrictions to use by non-academics: License needed. User should contact

Jan.Baumbach@CeBiTec.Uni-Bielefeld.DE.

Comment: Source code, all used datasets, and the clustering results can be obtained from the FORCE project website.

## Authors' contributions

TW and JB developed and implemented the heuristic FORCE. Together with FPL, TW and JB evaluated the data with ASTRAL95. JB integrated FORCE into CoryneRegNet. SR proposed to examine the clustering problem from the transitive graph projection viewpoint, modeled the similarity functions and supervised the whole project. All authors contributed to writing; and all authors read and approved the final manuscript.

## Appendix

### Algorithm 1 – Graph layouting

**Input: **similarity matrix (*S*_*ij*_)_1 ≤ *i<j *≤ *n *_with *S*_*ij *_: = *s*(*ij*) - *t*; circular layout radius *ρ*, attraction factor *f*_att_, repulsion factor *f*_rep_, number of iterations *R*

**Output: **node positions *pos *= (*pos*[1], ..., *pos *[*n*]); each *pos *[*i*] ∈ ℝ^2^.

1: *pos *= *arrangeAllNodesCircular*(*ρ*) ▷ initial layout

2: **for ***r *= 1 to *R ***do**

3:    ▷ Compute displacements Δ for iteration *r*

4:    initialize array Δ = (Δ [1], ..., Δ[*n*]) of displacement vectors to Δ[*i*] = (0, 0) for all *i*

5:    **for ***i *= 1 to *n ***do**

6:       **for ***j *= 1 to *i - *1 **do**

7:          **if ***S*_*i*, *j *_> 0 **then**

8:             *f*_*i *← *j *_= log(*d*(*i*, *j*) + 1)·*S*_*i*, *j*_·*f*_att _▷ attraction strength

9:          **else**

10:             *f*_*i *← *j *_= (1/log(*d*(*i*, *j*) + 1))·*S*_*i*, *j*_·*f*_rep _▷ repulsion strength

11:          Δ[*i*] + = *f*_*i *← *j*_·(*pos *[*j*] -*pos *[*i*])*/d*(*i*, *j*)

12:          Δ[*j*] - = *f*_*i *← *j*_·(*pos *[*j*] -*pos *[*i*])*/d*(*i*, *j*)

13:    ▷ Move nodes by capped displacement vectors

14:    **for ***i *= 1 to *n ***do**

15:       Δ [*i*] = (Δ [*i*]/||Δ[*i*]||)·min{||Δ[*i*]||, *M*(*r*)}

16:       *pos *[*i*] + = Δ [*i*]

17: **return ***pos*

### Algorithm 2 – Partitioning the layouted graph

**Input: **layout positions *pos*, initial and maximal clustering distances *δ*_init_, *δ*_max_, initial step size *σ*_init_, step size factor *f*_*σ*_, similarity matrix (*S*_*ij*_)_1 ≤ *i*<*j *≤ *n *_to compute costs

**Output: **best found *n *× *n *adjacency matrix E* describing a clustering, associated cost c*

1: *δ *= *δ*_init_, *σ *= *σ*_init_, c* = ∞, E* = (0)^*n *× *n*^

2: **while ***δ *≤ *δ *_max _**do**

3:    construct auxiliary graph *G*_*δ *_= (*V*, *E*_*δ *_) with *E*_*δ *_: = {*uv *: *d*(*u*, *v*) ≤ *δ *}

4.    detect connected components of *G*_*δ *_

5:    compute transitively closed adjacency matrix *E' *from *E*_*δ *_

6:    **if ***cost*(*E'*) <c* **then**

7:       E* = *E'*; c* = *cost*(*E'*)

8:    *σ *= *σ*·*f*_*σ*_; *δ *= *δ *+ *σ*

9: **return **(E*, c*)

## Supplementary Material

Additional file 1 Graph layout I. This file is an image illustrating the layout process of a graph with 41 nodes after (A) 3, (B) 10, and (C) 90 iterations. Click here for file

Additional file 2 Graph layout II. This file is an image illustrating the layout process of a graph with 10 nodes after (A) 3, (B) 10, and (C) 40 iterations. Click here for file

Additional file 3 Graphical clustering summary. This zipped file contains images summarizing the FORCE clustering results for the two similarity functions BeH and SoH, and all four datasets, similar to our Figure 1. We used MATLAB scripts provided by Paccanaro [7] to create these images. Click here for file

Additional file 4 Quality evaluation for different scoring themes and datasets. This file is tab-delimited and stores F-measures for a wide range of thresholds and coverage factors, for all used datasets and similarity functions. column 1: F-measure, column 2: coverage factor *f*, column 3: threshold, column 4: dataset/similarity function. Click here for file

Additional file 5 Quality evaluation for different thresholds and fixed coverage factor, dataset and similarity function. This file is tab-delimited and stores F-measures for a range of thresholds, and fixed coverage factor *f *= 20, dataset ASTRAL95_1_161, and similarity function BeH. Column 1: threshold, column 2: coverage factor *f*, column 3: F-measure. Click here for file

Additional file 6 Quality evaluation of Affinity Propagation for different scoring themes and datasets. This file is tab-delimited and stores F-measures for a wide range of parameter constellations and coverage factors, for all used datasets and similarity functions. Column 1: F-measure, column 2: coverage factor *f*, column 3: preference *pre*, column 4: damping factor *df*, column 5: dataset/similarity function. Click here for file
