# Supplementary figures and images for: Large scale clustering of protein sequences with FORCE -A layout based heuristic for weighted cluster editing
Source: BMC Bioinformatics. 2007 Oct 17;8:396. doi: 10.1186/1471-2105-8-396 (PMC2147039; doi:10.1186/1471-2105-8-396)

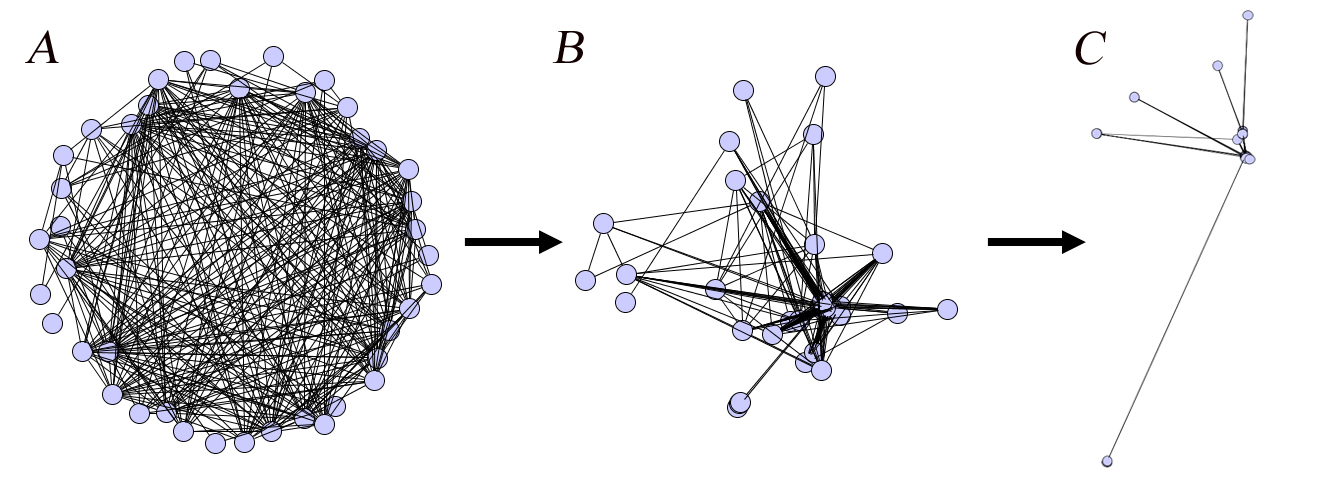

Supplement: Additional file 1 — Graph layout I. This file is an image illustrating the layout process of a graph with 41 nodes after (A) 3, (B) 10, and (C) 90 iterations. [file 1471-2105-8-396-S1.jpeg]

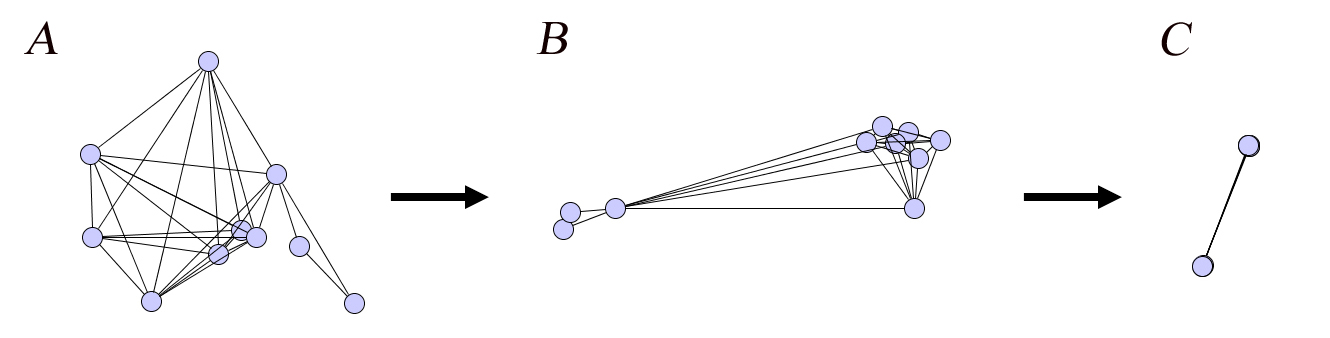

Supplement: Additional file 2 — Graph layout II. This file is an image illustrating the layout process of a graph with 10 nodes after (A) 3, (B) 10, and (C) 40 iterations. [file 1471-2105-8-396-S2.jpeg]

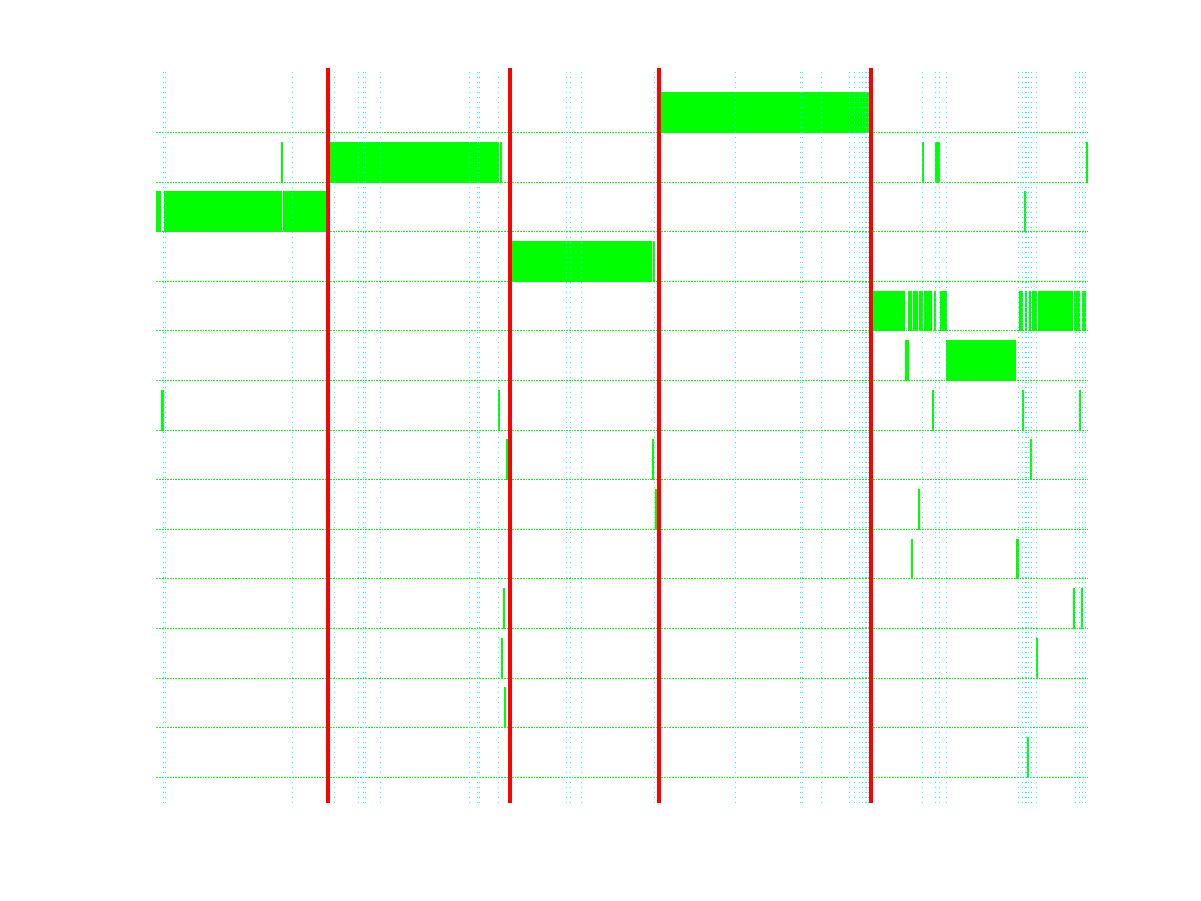

Supplement: Additional file 3 — Graphical clustering summary. This zipped file contains images summarizing the FORCE clustering results for the two similarity functions BeH and SoH, and all four datasets, similar to our Figure 1. We used MATLAB scripts provided by Paccanaro [7] to create these images. [file 1471-2105-8-396-S3.zip › ASTRAL95_1_171_SoH.jpg]

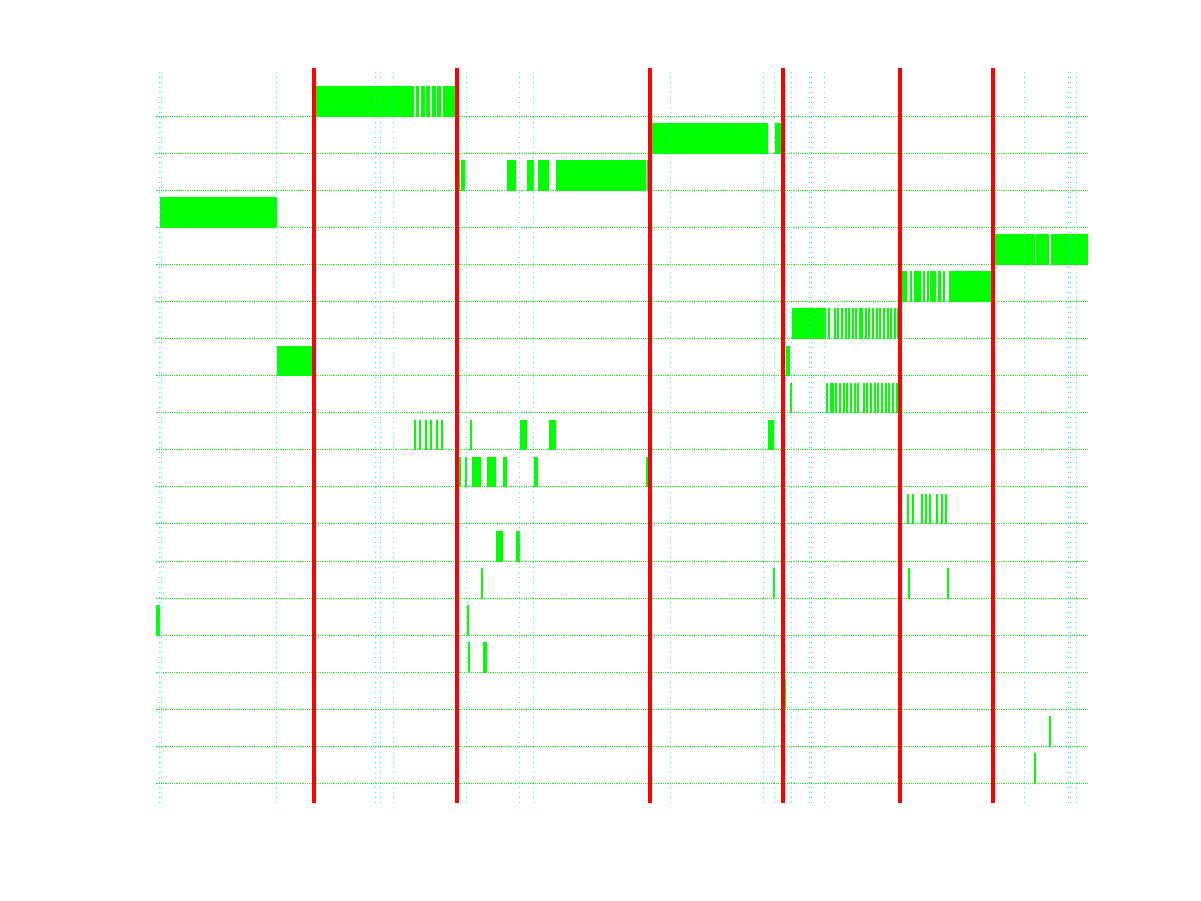

Supplement: Additional file 3 — Graphical clustering summary. This zipped file contains images summarizing the FORCE clustering results for the two similarity functions BeH and SoH, and all four datasets, similar to our Figure 1. We used MATLAB scripts provided by Paccanaro [7] to create these images. [file 1471-2105-8-396-S3.zip › ASTRAL95_2_161_BeH.jpg]

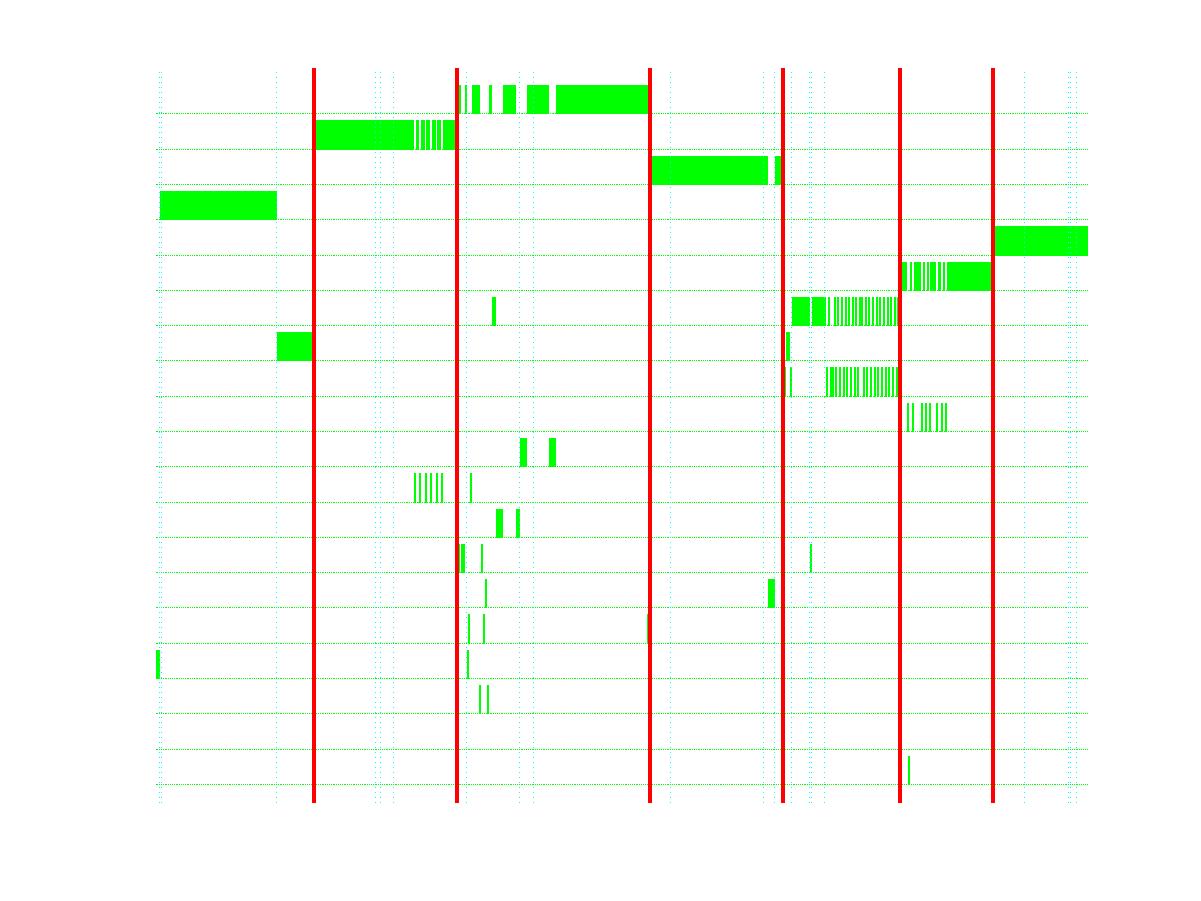

Supplement: Additional file 3 — Graphical clustering summary. This zipped file contains images summarizing the FORCE clustering results for the two similarity functions BeH and SoH, and all four datasets, similar to our Figure 1. We used MATLAB scripts provided by Paccanaro [7] to create these images. [file 1471-2105-8-396-S3.zip › ASTRAL95_2_161_SoH.jpg]

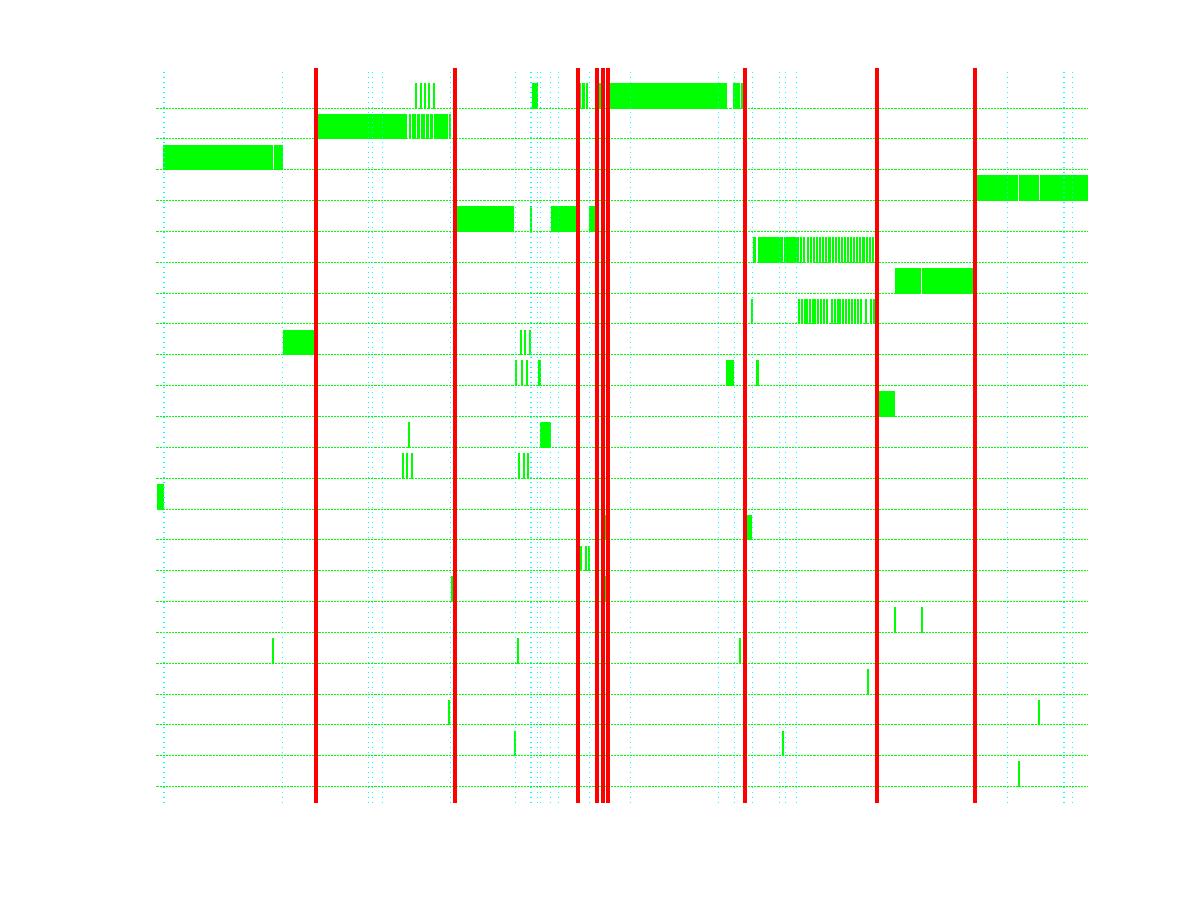

Supplement: Additional file 3 — Graphical clustering summary. This zipped file contains images summarizing the FORCE clustering results for the two similarity functions BeH and SoH, and all four datasets, similar to our Figure 1. We used MATLAB scripts provided by Paccanaro [7] to create these images. [file 1471-2105-8-396-S3.zip › ASTRAL95_2_171_BeH.jpg]

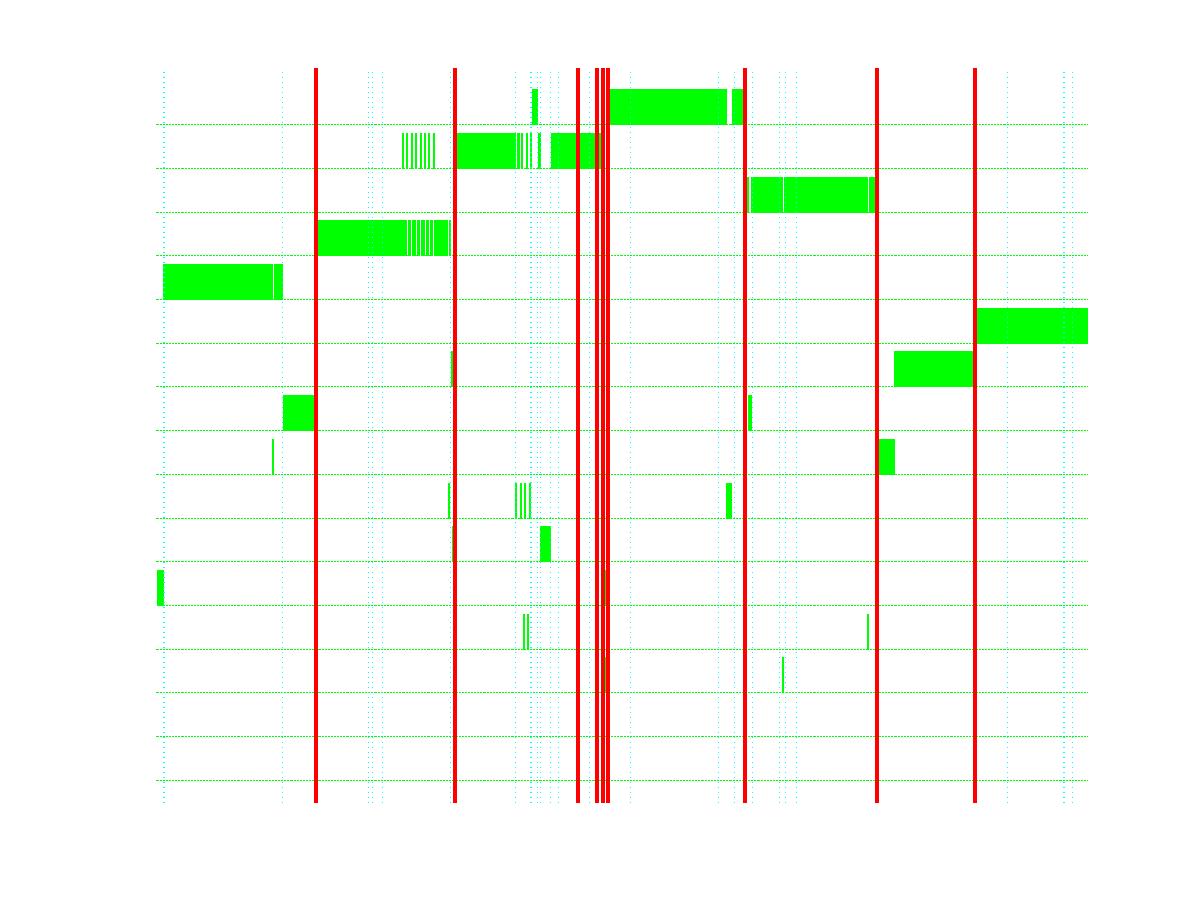

Supplement: Additional file 3 — Graphical clustering summary. This zipped file contains images summarizing the FORCE clustering results for the two similarity functions BeH and SoH, and all four datasets, similar to our Figure 1. We used MATLAB scripts provided by Paccanaro [7] to create these images. [file 1471-2105-8-396-S3.zip › ASTRAL95_2_171_SoH.jpg]

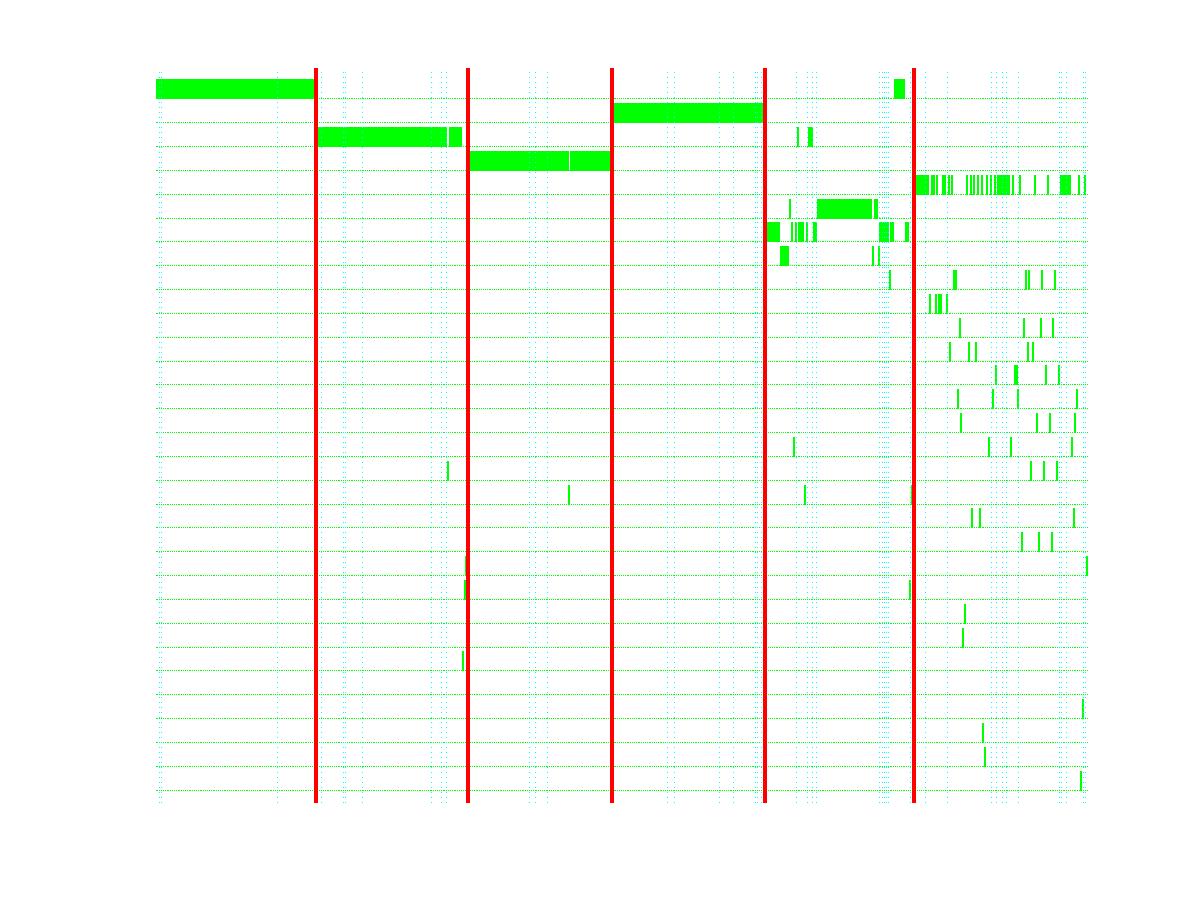

Supplement: Additional file 3 — Graphical clustering summary. This zipped file contains images summarizing the FORCE clustering results for the two similarity functions BeH and SoH, and all four datasets, similar to our Figure 1. We used MATLAB scripts provided by Paccanaro [7] to create these images. [file 1471-2105-8-396-S3.zip › ASTRAL95_1_161_BeH.jpg]

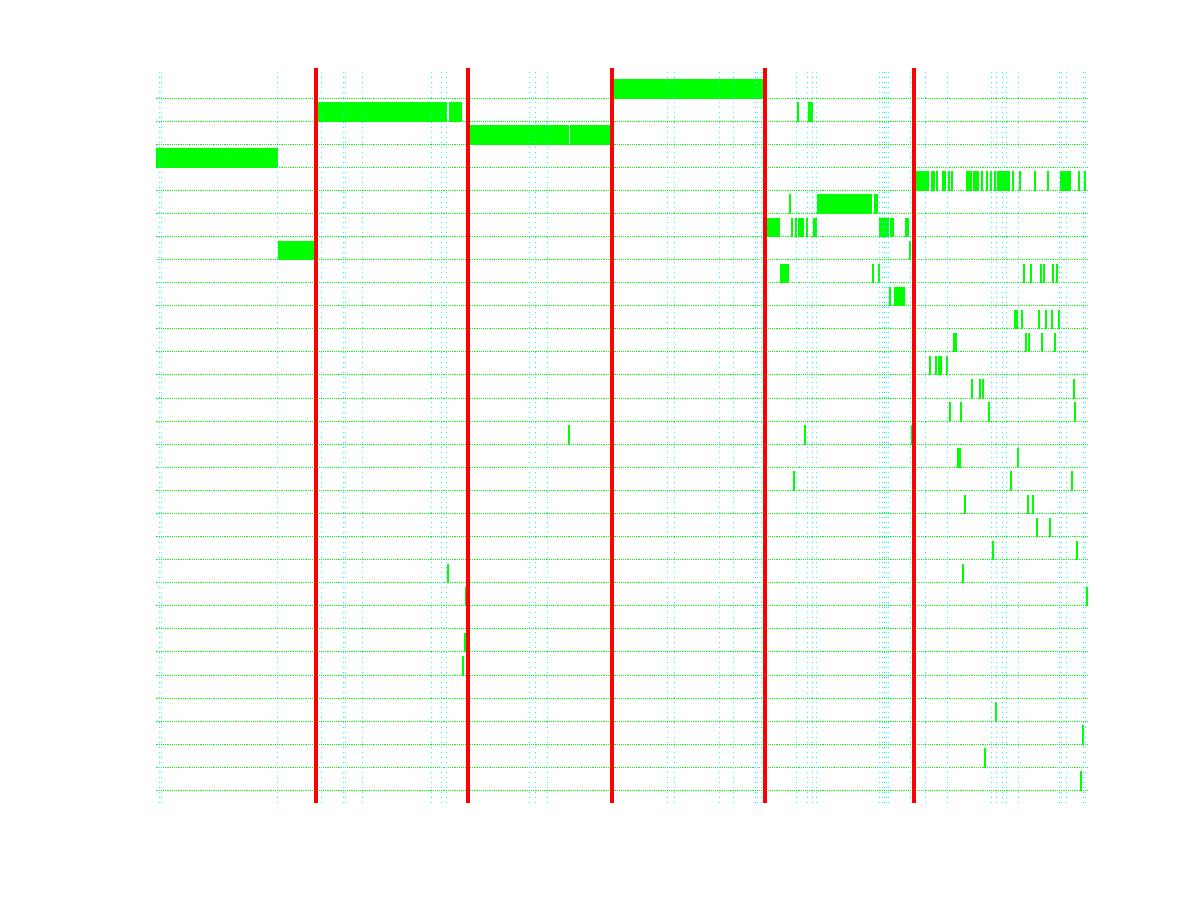

Supplement: Additional file 3 — Graphical clustering summary. This zipped file contains images summarizing the FORCE clustering results for the two similarity functions BeH and SoH, and all four datasets, similar to our Figure 1. We used MATLAB scripts provided by Paccanaro [7] to create these images. [file 1471-2105-8-396-S3.zip › ASTRAL95_1_161_SoH.jpg]

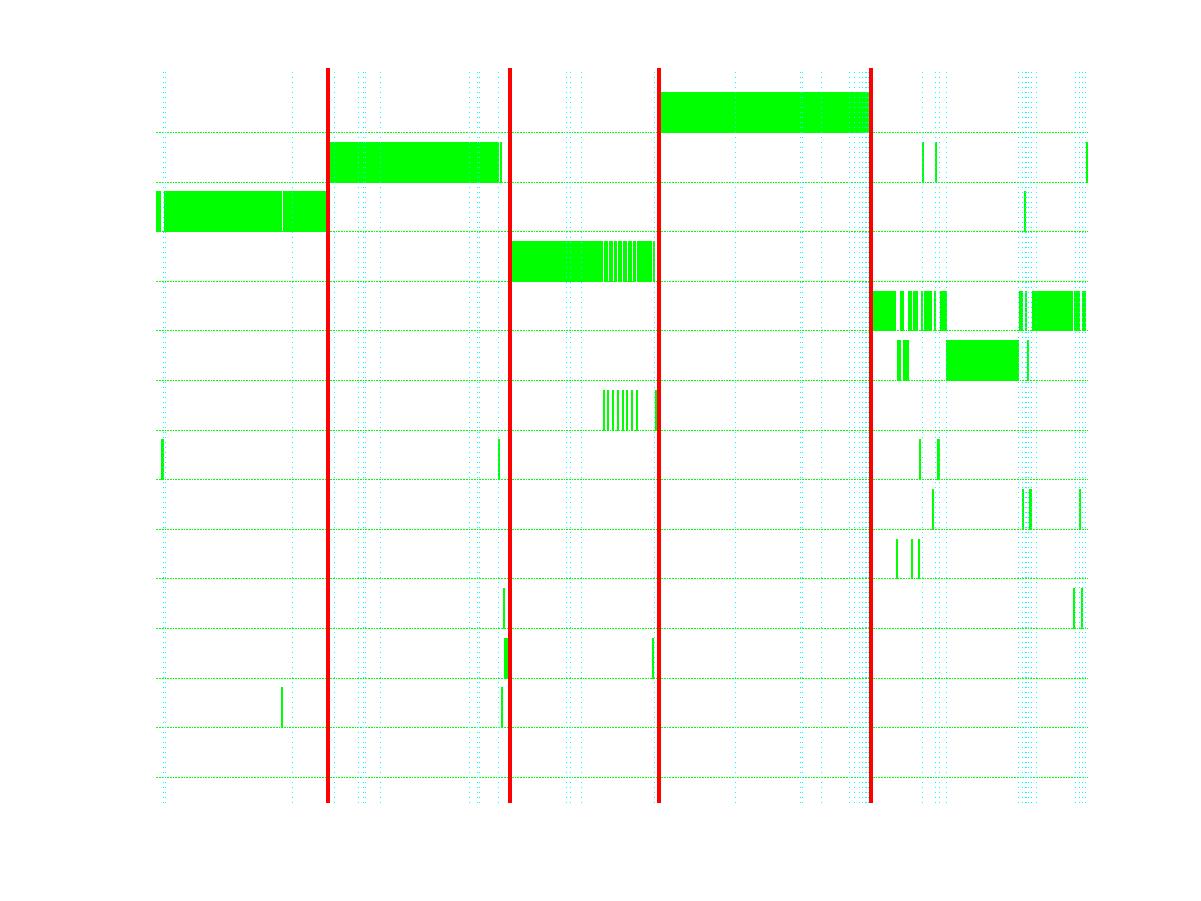

Supplement: Additional file 3 — Graphical clustering summary. This zipped file contains images summarizing the FORCE clustering results for the two similarity functions BeH and SoH, and all four datasets, similar to our Figure 1. We used MATLAB scripts provided by Paccanaro [7] to create these images. [file 1471-2105-8-396-S3.zip › ASTRAL95_1_171_BeH.jpg]
